# Closing the loop: the power of microbial biotransformations from traditional bioprocesses to biorefineries, and beyond

**DOI:** 10.1111/1751-7915.13713

**Published:** 2020-12-04

**Authors:** Paola Branduardi

**Affiliations:** ^1^ Department of Biotechnology and Biosciences University of Milano‐Bicocca Piazza della Scienza 2 Milano 20126 Italy

## Abstract

The power of microorganisms in manipulating diverse matrices and in favouring the flux of elements and molecules through biogeochemical cycles developed in the natural environment, but they also managed to take advantage of some niches created by humans. Therefore, inspired by learning these lessons from nature, we can implement biobased processes at industrial level, for diminishing our dependency on fossil resources and to return molecules to their turnover in a compatible timeframe and with reduced environmental impact.

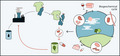

The decoupling between the economic (and demographical) growth rate and the availability of resources to sustain our production system is a fact (Sanyé‐Mengual *et al*., 2019). Similarly, there are scientific evidences underlying an interconnection (and consequences) between a polluted or corrupted environment and health (https://www.euro.who.int/en/health‐topics/environment‐and‐health), as well as reports asserting the anthropic responsibility of ‘opening the cycles of chemical elements’ (Finzi *et al*., 2011). Nevertheless, the myth of progress and linear growth is somehow very difficult to substitute in our mind and daily life, even because all‐pervasive alternatives are not available yet. On the other hand, every gap brings the potential of an opportunity. The linear model of growth is depleting fossil resources, as their turnover takes longer than their consumption. At the same time, it is generating large amounts of wastes, an abundant fraction of which are represented by organic wastes. This short‐sighted strategy also implies waste management and associated direct and indirect costs. In this challenging scenario, we as microbial biotechnologists are called (and willing!) to play a role. Unlocking the potential of microorganisms into industrial processes, we might offer the possibility to turn a problem (waste, pollution and lack of resources) into a solution (products, materials). This can happen because of the effectiveness of microorganisms in transforming molecules and in recycling chemical elements through biogeochemical cycles, known since the end of the XIX century, coupled with the more recent expertise on how to transform this tremendous potential into viable and competitive microbial‐based industrial processes. Microbial quantitative physiology, metabolic engineering, synthetic remodelling and process engineering are the main technologies that are innovating traditional fermentation processes, expanding the potential of biorefineries and allowing to reconsider the end of life of products and goods, with examples of industrial symbiosis. These processes aim at accomplishing some of the key tasks of the UN 2030 agenda in more than one of the 17 Sustainable Development Goals (SDG, https://sdgs.un.org/goals). We can mention SDG2 (zero hunger), SDG8 (decent work and economic growth), SDG9 (industry, innovation and infrastructure), SDG12 (responsible consumption and production) and SDG13 (climate action) among the more directly related ones, with an indirect but clear impact on others as well (https://biconsortium.eu/sites/biconsortium.eu/files/documents/Bioeconomy_and_the_SDGs_July%202018.pdf).

To exemplify these concepts here are some examples of prominent groups of microorganisms that are either confirming their supremacy in industrial processes, or are emerging as future bugs, in both cases revealing how their potential can help us to turn our linear model of production into biobased and circular processes.

## Fermented food and beverages: revisiting the ancient concept of preserving organic matrices

The establishment of production processes implying the generation of organic wastes occurred very early with the establishment of human social life style. However, when the production of food from raw materials took place, the economic model was still very close to the circularity of the system: residues were valorized in different ways, and proximity with natural cycles was real. The accurate use of resources was essential to ensure the survival of the community, and especially food became a way of creating the community and of sharing traditions (see for example, Anderson, 2014). One of the lessons that we have learnt from that time is that by fermentation, it is possible to expand the shelf life of many organic matrices, and artisanal tradition selected those processes resulting in enjoyable food and beverages with additional nutritional properties. Connected to that, microorganisms involved in indigenous food fermentation are often referred as the first organisms domesticated by humans. Nonetheless, if compared with animal and plant farming, the cultivation of microorganisms proceeded for much longer with a poor if not null knowledge of the biological principles behind. Even today, the exact knowledge of how some microbial consortia operate is object of studies as also demonstrated by the creation of libraries and collections (see, e.g. the world collection of sourdough, https://www.questforsourdough.com/puratos‐world‐heritage‐library).

The aims of current research are to expand the organoleptic and nutritional traits of the products, both for humans and animals, ensure food safety, enlarge the array of utilized substrates, and select novel robust cell factories or microbial consortia.

One prominent example is constituted by lactic acid bacteria (LAB), which are known to play a relevant role not only in determining the taste and the flavour of fermented products, but also in preserving and providing enhanced safety of food. This is possible because many LAB produce bacteriocins, bacteriostatic peptides released extracellularly that have antimicrobial activity, among other useful metabolites (Juturu and Wu, 2018). The multifaceted metabolisms of LAB have been investigated by omics‐based approaches, either on single species or on complex communities describing their impact on different fermented food products, which in addition of defining the quality of the products can have beneficial health effects (Şanlier *et al*., 2019). Moreover, globalization is allowing to expand the diffusion of typical dishes: ‐omics analyses on the one hand have the merit to provide quantitative data on the beneficial molecules that characterize these products, and on the other hand, they can describe microbial consortia able to utilize a variety of biomasses. As example, a recent study describes the LAB consortium responsible for the production of Kimchi, a traditional Korean dish made of salted and fermented vegetables (Lee *et al*., 2020).

Taken together, traits like a wide spectrum of fermentable substrates, an ample array of primary and secondary metabolite produced (Ashaolu, 2019), robustness in dominating over naturally occurring microflora as natural consortia, and tolerance against specific stressors of industrial interest (e.g., butanol, Li *et al*., 2010) make this clade very attractive not only to continue the innovation in fermented food, but also to expand their exploitation outside of the classical food sector (Sauer *et al*., 2017).

Moving from bacteria to yeasts, the prominent species responsible for fermented beverage and food production is represented by *Saccharomyces cerevisiae*. However, as demonstrated by the ‘1002 Yeast Genomes Project’ (http://1002genomes.u‐strasbg.fr/), many differences are hidden under the same name. Correlation between genotype and phenotype is at the basis of the divergence in metabolic activities that justifies the diverse products that can be obtained: leavening of sweet or salt doughs for bakery’s products; fermentation of grapes or cereals for wine or ALE beers. In reality, spontaneous fermentations usually start with a complex yeast community, but over time the nature of the fermented matrix and the microbial activity determine the definition of an environment that selects those yeasts that are tolerant to stresses, mainly organic acids and ethanol. This is how the fermentation ends with fewer species, with *S. cerevisiae* often emerging (Ganucci *et al*., 2018).

Nonetheless, despite being very robust and with a superior fitness in specific niches, *S. cerevisiae* does not have a wide range of substrates or temperature where it can be superior to other yeasts. This justify the insurgence and maintenance of hybrids, as in the case of *Saccharomyces pastorianus* in breweries and the supremacy of other yeasts for fermentation of different matrices, as in the case of *Kluyveromyces lactis* and raw milk. Remarkably, in these cases the anthropic effect is determinant and we are only now approaching the many implications of that.

In respect to *S. pastorianus*, its hybrid nature was only quite recently clarified (Libkind *et al*., 2011): the study of how the two parental strains, *S. cerevisiae* and *Saccharomyces eubayanus*, contribute to the superior capability of the hybrid to ferment wort efficiently at lower temperature allowed to depict the process leading to lager beer. At the same time, the newly discovered strains of the yeast *S. eubayanus* (referred as to ‘wild lager’) were used for producing and commercializing novel beers, and hybridization or targeted genome editing followed by selection of desired traits is pursued for creating more hybrids and strains that can contribute with different flavours to the production of novel alcoholic beverages (as recently reviewed in Gorter de Vries *et al*., 2019).

*K. lactis* is among the yeasts more largely utilized from ancient times to make cheese and kefir (Lachance *et al*., 2011). However, only very recently it was described how the ability of importing and catabolizing lactose was inherited accidentally from a dairy lineage of *Kluyveromyces marxianus* which itself had already evolved an existing transporter to favour import of lactose as a result of a selective pressure created by early human farmers when they started to convert raw milk into fermeted products (Varela *et al*., 2019). Considering that *K. lactis* is a well‐established industrial yeast and that *K. marxianus* is also approaching that stage, the description of the genetic basis and the molecular determinants responsible for lactose uptake and catabolism is crucial for better utilizing them as pure cultures or in consortia, even beyond food processing.

Robustness and substrate versatility are typical also of fungal fermentations: as agents involved in the production of many compounds, among which prominent examples are organic acids and enzymes, they can become the final product, as biomass enriched in protein (Finnigan *et al*., 2019). This is the case of the product named Quorn, where the specie *Fusarium venenatum* is typically grown in air lift bioreactor, but interesting studies are emerging where these single cell proteins can be produced by cultivating fungi in solid state fermentation on residual biomasses (see for a recent example the report on *Neurospora intermedia* by Gmoser *et al*., 2019).

Moving from artisanal indigenous fermentations towards a punctual description of the microorganisms and of the transformations led by their metabolisms is allowing a novel consciousness that can be translated into a better exploitation of organic matrices, in an accurate description and possible use of biodiversity. And, these notions can be extended beyond the sector of fermented food and beverages.

## Microbial‐based biorefineries: expanding the array of biobased products

The innovation of other classical microbial fermentations on the production of amino acids, preservatives, enzymes, natural antibiotics and high added value products (recombinant and pharma proteins, flavours, bioactive molecules) is reshaping our scenario and our market, even if in many cases the biotech contribution is not even evident to the general public. But still, the impact on a possible ‘emancipation’ from fossil‐based productions or the creation of truly sustainable value chains is limited. Either the volumes of the products are so little that microbial‐ and biobased solutions are conceptually relevant but do not have an impact on a large scale, or when productions are volumetrically relevant they mainly rely on the use of refined substrates. In the latter case, a further expansion of the market might create possible overlap, and therefore ethical concerns, with feedstock that should be addressed to the food chain. Therefore, the real turnabout comes when sustainable processes can face the challenge of very large volume products, which are fuels, chemical platforms and polymers.

The biorefinery can be considered as the crossroad of all these concepts and expectations. Defined as ‘the sustainable processing of biomass into a spectrum of marketable biobased products and bioenergy’ (http://task42.ieabioenergy.com/), and taking advantage from an established value chain as that of the pulp and paper industry (https://ec.europa.eu/growth/sectors/raw‐materials/industries/forest‐based/pulp‐paper_en), the biorefinery is intended to valorize biomasses into a wide range of products, not excluding high value‐added compounds but comprising large volumetric productions of low value‐added products such as biofuels and building blocks. For matching sustainability criteria, biorefineries have to avoid the use of the so‐called first‐generation biomasses (such as those edible, enriched in easily accessible sugars, in direct competition with the food chain), and move to second generation ones, constituted mainly by residual cellulose‐based, non‐edible biomass and agricultural waste or organic sidestreams of other production processes (such as crude glycerol, or whey). Another important criterion is the cascading: the diverse components of biomasses should be upgraded with as much added value as possible, and for the most appropriate application, depending on their initial and final market volume and value.

Biorefineries can process biomasses in different ways: thermal, chemical, physical and biological. In this context, microorganisms (and/or their enzymatic activities) are increasingly taking the scene, expressing their potential in combination with the other listed approaches or being the sole actors of valorization. Remarkably, microbial diversity can be matched with the uneven nature of the diverse biomasses, which in turn make possible the valorization of many kinds of residues, linking the biorefinery to the territory, geographically and economically speaking, with beneficial social implications as well.

Tracing numbers of operative biorefineries is not trivial, but it is undeniable that they are increasing, with a good degree of dispersion over territories. Most of them are in the commercial phase, followed by demo and R&D plants. This is the well depicted situation of biobased industry in Europe, where geographical differences and limited extension in lands is favouring the insurgence of a multifaceted scenario (https://datam.jrc.ec.europa.eu/datam/mashup/BIOBASED_INDUSTRY/index.html). It is more difficult to find out how many of these facilities are implementing the use of microorganisms or enzymes, even because in some cases they blend different technologies or are at the implementation stage.

In respect to microorganisms, well‐known workhorses as *Escherichia coli, Bacillus subtilis, Corynebacterium glutamicum* and *S. cerevisiae* (and in some cases lactobacilli) have been (and still are) extensively used in biorefineries based on first generation biomasses. Moving towards second generation biomasses, we assist at extensive cell factories’ engineering to accomplish process conditions and substrate composition (Wendisch *et al*., 2016), but at the same time we register the establishment of a wider array of cell factories. They are mainly selected for their natural abilities either to produce the product of interest from the different residual biomass [see for example *Lactobacillus diolivorans* on crude glycerol (Pflügl *et al*., 2014), *Haloferax mediterranei* on whey (Raho *et al*., 2020), *Rhodosporidium toruloides* on *Camelina sativa* meal hydrolysate (Bertacchi *et al*., 2020)], or for the innate robustness towards industrial constraints, such as physical parameters or chemicals, including inhibitory compounds released from biomasses or the high concentration of the final products. It is here important to mention that the production of biobased bulk chemicals or fuels has to occur with costs that are competitive in the market (van Dien, 2013). Therefore, another important criterion in choosing microorganisms is to obtain the highest titre, yield and productivity. To accomplish that, it is preferable to choose microorganisms displaying high carbon uptake rates with low biomass formation, or it is possible to apply bioprocess engineering as well as to implement synthetic circuits such as those related to quorum sensing (see for an overview on these concepts Wehrs *et al*., 2019).

Last but not least, microbial diversity can be exploited beyond the purpose of its natural evolution, and studied to design synthetic microbial communities capable of matching industrial requirements (Johns *et al*., 2016). This reinforces the concept that the transition towards sustainable biobased production cannot avoid to pass through a nature‐inspired design.

## The end of life of processes and products: can microorganisms inspire and teach us once more?

The concept of nature‐inspired design can be applied also to the end of biobased processes and to the end of life of products. If we manage to do so, then we can close the loop, and operate what in a life cycle assessment is called ‘from cradle to cradle’ approach.

In respect to the process, there can be cases in which biorefineries are valorizing entirely the feedstock into products, but there are other cases in which residual streams are obtained, either in the form of carbon and/or nitrogen enriched molecules, or in the form of gases or energies. These residues can be valorized by other plants, resembling the functionality of ecological systems. This is what is called industrial symbiosis (https://ec.europa.eu/environment/ecoap/about‐eco‐innovation/experts‐interviews/making‐industrial‐symbiosis‐business‐usual‐europes‐circular_en): it allows to establish an interconnected network which strives to mutual advantages creating a flux not only of energy and materials but also of technology. Industrial symbiosis is aimed at reducing environmental footprint and requirements of virgin raw materials. Considering that anthropic activities are having as direct and indirect effect the accumulation of C1 enriched off‐gases, a return to the cradle could be accomplished if we can use them to generate new organic molecules. Once more microorganisms can teach us many and diverse natural biochemical pathways for carbon fixation, that we are increasingly describing (Sánchez‐Andrea *et al*., 2020) and implementing in applied processes (Branduardi and Sauer, 2018). In addition to naturally autotrophic cell factories, known workhorses as *Pichia pastoris* can be also engineered for acquiring this metabolic trait (Gassler *et al*., 2020) and so reducing their dependence on organic carbon sources. Furthermore, carbon fixation through the Wood Ljungdahl pathway allows to obtain acetate, a key building block that can be efficiently redirected into the products of interest (Kiefer *et al*., 2020) and is promising to expand the current industrial application of this technology (Liew *et al*., 2016).

In respect to the end of life of products, it is desirable to augment the efficiency of natural occurring biodegradation, therefore accelerating the re‐entering of molecules and elements into the biogeochemical cycles. Recently, it was demonstrated that it is possible to design such a strategy also for recalcitrant polymers like the polyester polyethylene terephthalate (PET), as anthropic impact has created novel niches where bacteria have evolved and selected novel degrading enzymes (Yoshida *et al*., 2016). Tailored enzymatic cocktails are currently in the implementation stage at industrial scale (https://carbios.fr/en/technology/biodegradation/).

## Conclusions

Overall, it is evident that microbial biotechnology has the potential and the power to play a pivotal role in promoting an effective industrial transition towards biobased processes of production, along the whole value chain. Because biobased processes aim at zero waste, prolonged life cycle of products, and regenerating resources, which are all pillars of the circular economy vision, Industrial Biotechnology is indicated as one of the Key Enabling Technologies that is proving to bring long‐term sustainability and growth in various economic sectors.

Would these premises and the successful stories already on the market also promote a change of paradigm towards an effective sustainability at social level and in our behaviour? Maybe. As humans, very likely with different degrees of consciousness or perception, we took a distance from nature. For many of us, we command the hierarchy of living organisms, and the expression of being a vegetable is intended to be offensive. Microorganisms, arthropods and invertebrates are dynamically modelling organic and inorganic matrices therefore promoting and sustaining life on our planet. Despite this, most of us would feel uncomfortable at the idea of lying in direct contact with soil, or even many of us never experienced the joy of manipulating humid soil with hands. This without knowing that for the principle of reciprocity, microorganisms can positively affect our life but the anthropic impact can have a negative effect on microorganisms, which in turn is going to have consequences for us (Cavicchioli *et al*., 2019). In this respect, in addition to being engaged with our science, as microbiologists we have the responsibility to introduce these concepts into our teaching. Teaching can be intended to specifically train scientists, but also to create a reciprocal exchange among stakeholders by integrating life science with social science concepts. This might be done at different levels, as in flexible courses (Sacchi *et al*., 2020), willing to promote a novel and shared vision of our wellbeing as embedded in our living planet, and therefore guiding with more consciousness our society towards biobased innovation.
